# Extending Prayer Marks as a Sign of Worsening Chronic Disease

**DOI:** 10.3329/jhpn.v29i3.7877

**Published:** 2011-06

**Authors:** M. Cangiano, Mohammod J. Chisti, Mark A.C. Pietroni, Jonathan H. Smith

**Affiliations:** ^1^University of Vermont College of Medicine, Burlington, VT 05405, USA; ^2^ICDDR,B, GPO Box 128, Dhaka 1000, Bangladesh; ^3^Portex Unit: Paediatric Anaesthesia, UCL Institute of Child Health, London, UK

**Keywords:** Chronic disease, Elbows, Extending, Prayer marks, Bangladesh

## Abstract

A 60-year old Muslim man was admitted to the Dhaka Hospital of ICDDR,B with an exacerbation of his chronic obstructive pulmonary disease. Incidental hyperpigmented skin lesions were noticed overlying the dorsum of his ankles, knees, and elbows. Such asymptomatic areas of thickened, lichenified and hyperpigmented skin are called ‘prayer marks’ and are well-imprinted on the knees, ankles, and forehead. These are secondary to prolonged periods of pressure over bony prominences during prayer. The patient's wife stated that the appearance of the elbow marks had coincided with an increase in his breathlessness and subsequent use of his elbows to rise from daily prayers. Prayer marks extending to the elbows could be a sign of worsening chronic disease.

## INTRODUCTION

One of the five pillars of Islam is ‘Sallah’ which is the ritualized performance of prayers five times a day. During ‘Sallah’, the devotee must perform ‘Sajdah’ which is a repeated prostration of the body involving the placing of the bare forehead, palms of hands, both knees, and the base of the toes upon the ground. These repeated and prolonged periods of pressure can lead to the appearance of asymptomatic, thickened, lichenified and hyperpigmented areas of skin. The usual distribution of prayer marks is on the forehead, elbows, knees, and ankles (1). The extension of prayer marks on to the elbows, to our knowledge, has not previously been described.

## CASE REPORT

A 60-year old man presented to the Dhaka Hospital of ICDDR,B with an increase in shortness of breath. He had previously been diagnosed with chronic obstructive airways disease (COPD) for which he was taking inhaled salbutamol as required. On examination, he was found to be febrile, tachypnoeic and had widespread, coarse and fine crackles with added wheeze throughout both lung fields. His oxygen saturations were 80% on air and 92% on six litre per minute of oxygen via a face-mask. In addition, he was found to be tachycardic and hypotensive; on auscultation, there was a third heart sound, and bilateral pitting oedema was present to the level of the mid-shin. A provisional diagnosis of ‘acute exacerbation of COPD complicated by biventricular failure’ was made. Initial treatment included intravenous antibiotics, oxygen, hydrocortisone, nebulized salbutamol, frusemide, and a dopamine infusion. Subsequent management included aminophylline and captopril which was successful, and the patient was discharged from the hospital.

**Fig. 1.**
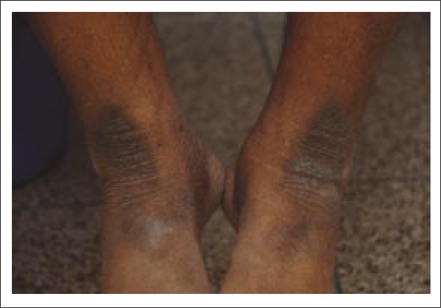
Prayer marks at ankles

**Fig. 2.**
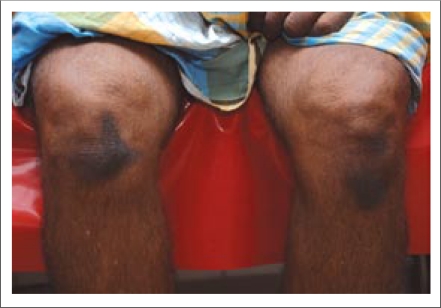
Prayer marks at knees

**Fig. 3.**
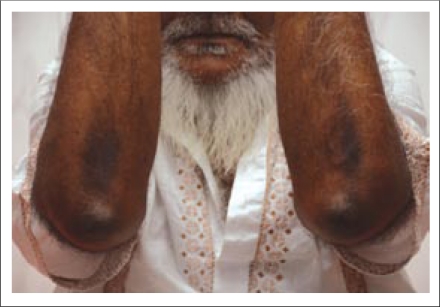
Prayer marks on elbows

During the treatment of the patient, hyperpigmented skin lesions were noticed overlying the dorsum of his ankles, knees, and elbows (Fig. 1-3). On questioning of the patient's wife, it was revealed that the appearance of the markings on the elbows had occurred over at least the last six months and coincided with a severe deterioration of his COPD. As a result of the patient's increased breathlessness on exertion, he was using his elbows to help himself rise from his daily prayers.

## DISCUSSION

The taking of a clear and detailed history is an essential part of any medical assessment. History-taking should not be confined to the initial consultation but should be an ongoing process of learning—both about the patient and how they deal with the disease process during their daily life.

Salient points of the history may also be learnt from close family members as they have the advantage of viewing changes over time in a more objective manner. In this case, the patient's wife had noted the skin changes on his elbows (Fig. 3) over the last six months and had associated their appearance with a deterioration of his exercise tolerance.

### Conclusion

The skin lesions on his elbows were an extension of his prayer marks into a position that is not normally described. Once noted by a thorough clinical examination, such a change in skin-markings may be a sign of worsening chronic disease, and the taking of a careful history may reveal the temporal course of such deterioration.

## References

[1] Abanmi AA, Al Zouman AY, Al Hussaini H, Al-Asmari A (2002). Prayer marks. Int J Dermatol.

